# High frequency of CD29^high^ intermediate monocytes correlates with the activity of chronic graft-*versus*-host disease

**DOI:** 10.1111/ejh.12160

**Published:** 2013-07-12

**Authors:** Masahiro Hirayama, Eiichi Azuma, Shotaro Iwamoto, Keishiro Amano, Atsuko Nakazawa, Shigehisa Tamaki, Eiji Usui, Yoshihiro Komada

**Affiliations:** 1 Department of Pediatrics and Cell Transplantation, Mie University Graduate School of MedicineTsu, Mie, Japan; 2 Division of Pathology, Department of Clinical Laboratory Medicine, National Center for Child Health and DevelopmentSetagaya, Tokyo, Japan; 3 Department of Internal Medicine, Ise Red Cross HospitalIse, Mie, Japan

## *To the Editor*:

Chronic graft-*versus*-host disease (cGVHD) is a major cause of morbidity and mortality in patients after allogeneic hematopoietic stem cell transplantation (HSCT) 1–2. ‘Trial-and-error system’ remains the only way to identify the effectiveness of immunosuppressive drug in the individual patient, and valid biomarkers for cGVHD are eagerly needed to identify the response to the drug 3–4. Monocyte-derived interleukin-10 (IL-10) spot-forming cells (SFCs) can be used as a biomarker for evaluating the activity of cGVHD 5. Recently, monocytes have been classified into three subpopulations 6. Among them, CD14^++^CD16^+^ intermediate monocytes are found at low frequency, but they have unique features and expand with cytokine treatment and in inflammation 7. Now, we have demonstrated that CD29^high^ intermediate monocytes increased in cGVHD. These results could be clinically relevant for treatment strategies.

We have updated our published results 5 in this study by adding thirty additional samples from 57 previous and three new patients for flow cytometric analysis because a new standard of monocyte phenotypes was reported 6. Patients were subclassified into those with no, active, and inactive cGVHD as previously described 5,8. Peripheral blood mononuclear cells (PBMCs) were obtained from patients who underwent allogeneic HSCT between 2000 and 2012 with no evidence of infection. Fresh samples were obtained and assayed from patients between 6 and 139 months (mean: 47.9 months) after HSCT. The study was registered (UMIN-Clinical Trials Registry: 000006733).

**Figure 1:**
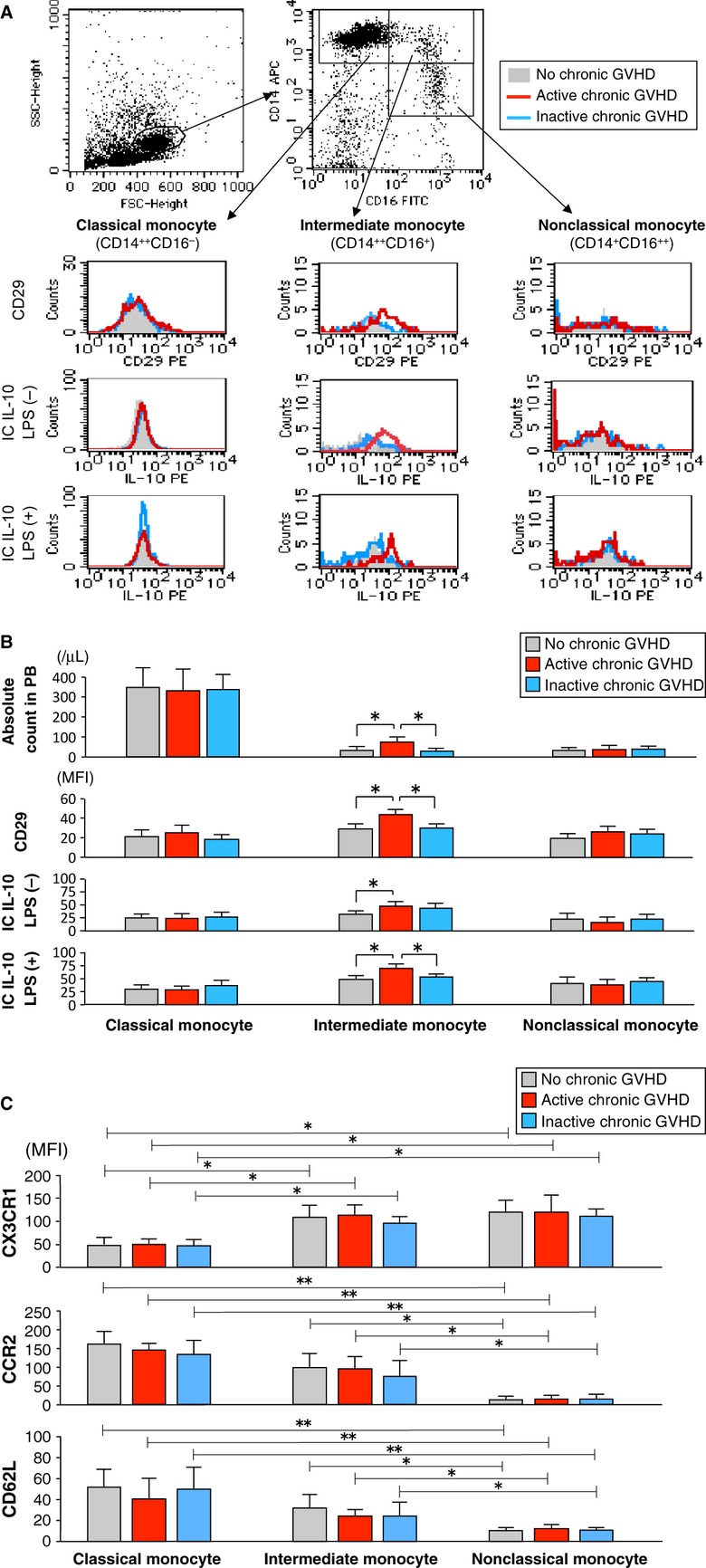
CD29^high^ intermediate monocytes produced IL-10 in patients with active chronic graft-*versus*-host disease (GVHD). (A) Representative dot plots gated with monocytoid light-scatter characteristics were classified into three subsets of monocytes. Each subset of monocytes was gated and identified for the expression of CD29 and intracellular (IC) IL-10 in histogram plots. Gray area indicates expressions in patients with no chronic GVHD; red line, active chronic GVHD; blue line, inactive chronic GVHD. For IC IL-10 staining, PBMCs were stimulated with or without lipopolysaccharide (LPS) (1 μg/mL). (B) Absolute counts in peripheral blood (PB) and mean fluorescence intensity (MFI) of CD29 and IC IL-10 for three subsets of monocytes were compared in patients with no chronic GVHD (*n* = 10), active chronic GVHD (*n* = 10), and inactive chronic GVHD (*n* = 10). Data were expressed as mean ± SD. *P < 0.05. (C) MFI of each subset of monocytes for CX3CR1, CCR2, and CD62L was compared with the corresponding state of chronic GVHD activity in patients with no chronic GVHD (*n* = 10), active chronic GVHD (*n* = 10), and inactive chronic GVHD (*n* = 10). Data were expressed as mean ± SD. *P < 0.05. **P < 0.01.

First, we have performed flow cytometric analysis of the expression of CD29, a ligand of fibronectin, and intracellular (IC) IL-10 in monocytes after stimulation with or without lipopolysaccharide (LPS) in patients after HSCT (Fig. 1A). The absolute number of intermediate monocytes, but neither classical nor nonclassical monocytes, increased significantly in patients with active cGVHD compared with those with no or inactive cGVHD (*P* < 0.05) (Fig. 1B). The expression of CD29 on intermediate monocytes was significantly higher (*P* < 0.05) in patients with active cGVHD than those with no or inactive cGVHD. CD29 expression of monocytes increased after reacting with fibronectin as we have reported 5. Fibronectin strongly deposited under the basal layer of skin in cutaneous cGVHD 5–10. Moreover, the expressions of IC IL-10 on intermediate monocytes from patients with active cGVHD stimulated both with and without LPS were significantly higher (*P* < 0.05) than those with no or inactive cGVHD (Fig. 1B). These results suggest that CD29^high^ intermediate monocytes were the main producer of IL-10 during active cGVHD.

Secondly, we studied chemokine/homing receptors on monocytes. Classical monocytes represented with lower expression of CX3CR1 (*P* < 0.01) and higher expression of CCR2 (*P* < 0.01) and CD62L (*P* < 0.05) than each expression of nonclassical monocytes (Fig. 1C). Intermediate monocytes showed intermediate expressions for CX3CR1, CCR2, and CD62L between classical and non-classical monocytes. Namba *et al*. 11 suggested that CX3CR1^+^ monocytes might be recruited from the circulation to the fractalkine^+^ epidermis in cGVHD. CCR2 is a receptor for monocyte chemoattractant protein-1 and it involved in migration of monocytes to inflammatory site. CD62L has a key role in adherence of monocytes to vascular endothelium from the blood 12. Thus, migration of blood monocytes into target organ of cGVHD might be controlled by chemokine/homing receptors.

In summary, the findings described here have demonstrated that flow cytometric analysis of intermediate monocytes can be used, as a simple and useful biomarker, for monitoring the activity of cGVHD. When patients enter into non-active cGVHD clinically, intermediate monocytes may be used for when to quit immunosuppressants properly.
